# Relationship between rs854560 *PON1* Gene Polymorphism and Tobacco Smoking with Coronary Artery Disease

**DOI:** 10.1155/2017/1540949

**Published:** 2017-09-29

**Authors:** Joanna Iwanicka, Tomasz Iwanicki, Paweł Niemiec, Tomasz Nowak, Jolanta Krauze, Władysław Grzeszczak, Sylwia Górczyńska-Kosiorz, Anna Ochalska-Tyka, Iwona Żak

**Affiliations:** ^1^Department of Biochemistry and Medical Genetics, School of Health Sciences in Katowice, Medical University of Silesia, Medyków Street 18, 40-752 Katowice, Poland; ^2^1st Department of Cardiac Surgery/2nd Department of Cardiology, American Heart of Poland, S. A. Armii Krajowej Street 101, 43-316 Bielsko-Biala, Poland; ^3^Department of Internal Medicine, Diabetes and Nephrology, School of Medicine and Division of Dentistry in Zabrze, Medical University of Silesia, 3 Maja Street 13-15, 41-800 Zabrze, Poland; ^4^Regional Center of Blood Donation and Blood Treatment in Racibórz, Sienkiewicza Street 3, 47-400 Racibórz, Poland

## Abstract

Paraoxonase-1 (PON1) is the antioxidant marker of high-density lipoproteins protecting against atherosclerosis and coronary artery disease (CAD) phenotype. The purpose of the present study was to determine whether the *PON1* gene rs854560 polymorphism (163T>A) is associated with CAD in Polish population. rs854560 was genotyped in 494 subjects: 248 patients with premature CAD and 246 blood donors as a control. We found that the risk of CAD was significantly higher in TT homozygotes than in A allele carriers (OR = 1.87, *p* = 0.041). The synergistic effect between the TT genotype and cigarette smoking was observed (SIM = 9.81; SI = 14.70). The relative increase in risk from interaction between factors was over 37 (RERI = 36.13). The *PON1* polymorphism did not modulate the risk of CAD in response to exposure to other traditional risk factors. In conclusion, the rs854560 polymorphism may modulate the risk of CAD in response to cigarette smoking in Polish population. Carriers of TT genotype seem to be particularly at risk of CAD, when exposed to cigarette smoking.

## 1. Introduction

Coronary artery disease (CAD) is one of the main causes of death worldwide. Oxidative modification of low-density lipoproteins (LDL) in the vessel wall may play a major role in development of atherosclerosis, which can progress to CAD. Atherosclerosis is characterized by the inflammation build-up of fatty lesions and scarring of arterial walls with oxidative stress as a primary contributing factor [1]. Atherosclerosis is the main reason of CAD which has a multifactorial background and results from numerous interactions between genetic and environmental factors. Lipid abnormalities, cigarette smoking, overweight/obesity, male gender, and older age are the main traditional risk factors for CAD. High-density lipoproteins (HDL) protect against CAD, and the paraoxonase 1 (PON1) enzyme provides their main source of antioxidative activity.

PON1 is a calcium-dependent enzyme, responsible for the hydrolysis of oxidized phospholipids within LDL. Its action prevents the accumulation of oxLDL and protects against the initiation and progression of atherosclerosis [2–5]. PON1 is synthesized in the liver and is secreted to the bloodstream where it bounds HDL through the A1 and J apoproteins [6]. It increases the enzyme stability [7].

Low levels, or the complete absence, of PON1 reduce the antioxidative properties of HDL [8]. Therefore, loss of PON1 enzymatic activity can promote the manifestation of atherosclerosis and CAD phenotypes.

PON1 is encoded by the *PON1* gene (7q21.3–22.1) [9], which contains approximately 200 single nucleotide polymorphisms [10], including the 163T>A polymorphism (rs854560). The rs854560 results in the substitution of methionine to leucine at amino acid residue 55 (L55M) of the PON1 polypeptide [11]. Several studies have shown that this *PON1* variant could play an important role in Parkinson's disease [12], bipolar I disorder [13], ovarian cancer [14], breast cancer [15], and prostate cancer [16]. Moreover, some but not all previous studies have linked this polymorphism with CAD [11, 17–21]. The selection of the 163T>A polymorphism for the study was due to its functional significance (influence of alleles and genotypes on PON1 activity), and the fact that its role in predisposition to CAD is not entirely clear (discrepant results of previous case-control studies). Additionally, we searched for potential interactions between *PON1* alleles and common traditional risk factors of CAD including cigarette smoking, hypertension, and plasma lipid abnormalities.

## 2. Materials and Methods

### 2.1. Subjects

We enrolled 494 Polish Caucasians in our case-control study. All participants were inhabitants of the Upper Silesia region. The patient group included individuals with angiographically confirmed premature CAD (75 females and 173 males), aged 44.58 ± 5.98 years. The control group consisted of 246 blood donors with negative familial history of CAD, aged 43.58 ± 6.32 years. CAD subjects were recruited by the same clinician from the First Department and Clinic of Cardiology at the Upper Silesian Center of Cardiology in Katowice and the First Department of Cardiac Surgery at the Upper Silesian Center of Cardiology in Katowice. Controls were selected from blood donors of the Regional Centers of Blood Donation and Blood Treatment in Katowice and Racibórz.

Inclusion and exclusion criteria, details of the medical interview, diagnosis and evaluation, criteria for CAD, myocardial infarction, and risk factors were as described previously [22].

This study was approved by the Ethics Committee of the Medical University of Silesia in Katowice (Poland) and informed written consent was obtained from all subjects.

### 2.2. Serum Lipid Measurement

Plasma total cholesterol (TC), HDL cholesterol, and triglyceride (TG) levels were obtained by enzymatic colorimetric methods (Analco, Warsaw, Poland). LDL cholesterol levels were calculated using the Friedewald formula [23].

### 2.3. DNA Extraction and Genotyping

Genomic DNA was extracted from peripheral leukocytes using the MasterPure genomic DNA purification kit (Epicentre Technologies, Madison, WI, USA). *PON1* polymorphisms were genotyped using the TaqMan® Pre-designed SNP Genotyping Assay Kit (Applied Biosystems, Foster City, CA, USA). The 20 *μ*L reaction mix consisted of the following: 1 *μ*L template DNA (15 ng/*μ*L), 10 *μ*L TaqMan Genotyping Master Mix (cat. number 4371355), 1 *μ*L probe (TaqMan Pre-designed SNP Genotyping Assay), and 8 *μ*L deionized water. The probe was diluted in TE buffer (10 mM Tris-HCl (pH 8.0) 0.1 mM EDTA) (1 : 1) before the reaction. PCR was performed according to the manufacturer's specifications. Genotyping was performed using a 7300 Real-Time PCR System (Applied Biosystems). Genotyping was successful in 92% of participants. Genotyping accuracy was checked by regenotyping 15% of the samples, and the reproducibility of the results was 100%.

### 2.4. Statistical Analysis

Data were analysed using *Statistica 12.0* (STATSOFT, Tulsa, OK, USA) and *SAS 9.1* (SAS Institute Inc., NC, USA) software. The Shapiro-Wilk test was used to check the normality of distribution. Comparison of quantitative data was performed by Mann–Whitney *U* test (nonnormal distribution) or the Student *t*-test (normal distribution). Allele frequencies were deduced from the genotype distributions. Hardy-Weinberg equilibrium testing, comparisons of genotypes, and allele frequencies between cases and control subjects were compared by a *χ*^2^ test. Statistical significance was accepted at *p* < 0.05. Odds ratios (OR with 95% confidence intervals) were computed using univariate and multiple logistic regression analyses after adjustment for age, gender, and traditional CAD risk factors. When the number of individuals in any of the analysed subgroups was zero, risk ratio values (95% CI) were used.

To identify potential biological interactions between *PON1* genotypes and traditional CAD risk factors, the 4 × 2 table approach was used. Subjects without the risk allele/genotype, not exposed to specific traditional risk factors, were used as a reference group (00 code). This group was compared to subgroups of subjects exposed to only traditional risk factors, only genetic risk factors, and to both traditional and genetic risk factors (codes 01, 10, and 11, resp.). Synergy measures in the multiplicative and additive models were used to interpret the amount of interaction, according to recommendations in the literature [24, 25]. Synergy indexes were calculated on the basis of OR values from the 4 × 2 tables, using the following formulas:

(i) For SIM (multiplicative synergy index),
  (1) $$\begin{array}{c} SIM=\frac{{OR}_{11}}{{OR}_{01}\times {OR}_{10}}. \end{array}$$

The ranges of SIM are from 0 to +∞. SIM < 1 means negative interaction (antagonism), SIM = 1 means no interaction, and SIM > 1 means positive interaction (synergy).

(ii) For SI (Rothman's additive synergy index),
  (2) $$\begin{array}{c} SI=\frac{{OR}_{11}-1}{\left({OR}_{01}-1\right)+\left({OR}_{10}-1\right)}. \end{array}$$

The ranges of SI and their interpretation are the same as in the case of SIM.

The RERI (relative excess risk due to interaction) parameter represents the relative risk increase resulting from interaction between factors, and the AP (proportion attributable to interaction) of the combined effect due to interaction was also calculated, using the following formulas [24]:

(3) $$\begin{array}{c} RERI={OR}_{11}-{OR}_{10}-{OR}_{01}+1. \end{array}$$

The ranges of RERI vary from −∞ to +∞. RERI < 0 means negative interaction, RERI = 0 means no interaction, and RERI > 0 means positive interaction.

(4) $$\begin{array}{c} AP=\frac{RERI}{{OR}_{11}}. \end{array}$$

The ranges of AP are from −1 to +1. AP < 0 means negative interaction, AP = 0 means no interaction, and AP > 0 means positive interaction.

Asymmetric confidence intervals (CI) for additive interaction parameters (SI, RERI, and AP) were determined using the model of Zou [26].

## 3. Results

### 3.1. Study Group Characteristics

The characteristics of CAD patients and blood donors are shown in Table 1. CAD patients had increased total cholesterol, LDL cholesterol, and triglyceride levels and higher body mass index values. Furthermore, the level of HDL cholesterol was significantly lower in CAD patients (Table 1).

### 3.2. Analysis of the rs854560 *PON1* Gene Polymorphism

The genotype and allele frequencies of the rs854560 polymorphism are shown in Table 2. All genotype frequencies conformed to Hardy-Weinberg equilibrium. The frequency of the TT genotype was higher in the CAD group than in the blood donor group (*p* = 0.041). The risk of CAD was almost two times higher in TT homozygotes than in A allele carriers. This association was not confirmed in the multivariate model after adjustment for traditional risk factors of CAD.

### 3.3. The rs854560 *PON1* Gene Polymorphism and Clinical CAD Phenotypes

There were no statistically significant differences between rs854560 polymorphism genotypic variants and myocardial infarction, severe atherosclerosis (presence of multivessel coronary disease or critical occlusion >90%) observed during coronary angiography, left ventricular hypertrophy, and diabetes mellitus.

### 3.4. Interactions between rs854560 *PON1* Genotypes and Traditional CAD Risk Factors

Potential interactions between the respective rs854560 genotypes and traditional CAD risk factors (male gender, cigarette smoking, hypertension, diabetes mellitus, overweight/obesity, and plasma lipid abnormalities) were analysed.

The synergistic effect was observed between the TT genotype and cigarette smoking (Table 3). Cigarette smokers with the TT genotype had an increased risk of CAD than did smokers with the A allele (AA + AT) and nonsmokers with the TT genotype.

When analysing the relationship between cigarette smoking and the rs854560 phenotype using the multiplicative model of interaction, the estimated risk of CAD was almost 10 times higher than expected (SIM = 9.81, 95% CI: 1.11–86.97, *p* = 0.04). When analysing the same interaction using the additive model of interaction, the estimated risk of CAD was almost 15 times higher than expected (SI = 14.70, 95% CI: 1.74–123.98). In addition, the relative increase in risk from interaction between factors was over 37 (RERI = 36.13, 95% CI: 1.76–296.93) and the value of proportion of the combined effect resulting from interaction was 0.91 (0.91, 95% CI: 0.13–0.98) (SI = 14.70, 95% CI: 1.74–123.98).

The *PON1* polymorphism did not modulate the risk of CAD in response to exposure to other traditional risk factors such as the following: male gender, hypertension, diabetes mellitus, overweight/obesity, and plasma lipid abnormalities (Table 4). Hypertension and diabetes mellitus were excluded from the analysis because of lack of these phenotypes in the control group (factors that prevent being a blood donor in Poland).

### 3.5. Analysis of the rs854560 *PON1* Gene Polymorphism Depending on Smoking Status

To evaluate whether the *PON1* polymorphism may be a determinant of smoking habit, we divided our entire group into smokers and nonsmokers. The frequencies of genotypes and alleles are shown in Table 5. There were no differences in genotypic variant distribution between smokers and nonsmokers (Table 5).

## 4. Discussion

The multifactorial nature of CAD results from interactions between genetic and environmental factors. The *PON1* rs854560 polymorphism potentially modifies the risk of CAD. Here, we show that rs854560 polymorphism TT homozygosity predisposes individuals to CAD in the Polish population.

Association between the TT rs854560 genotype and CAD may be explained by the effects of polymorphism variants on the activity of paraoxonase 1. The presence of leucine at amino acid residue 55 of the PON1 polypeptide (encoded by the *PON1* A allele) is associated with increased enzymatic activity compared to the 55M phenotype (encoded by the *PON1* T allele) [27]. Furthermore, the lowest PON1 activity was observed in 55MM, TT homozygotes [28]. In the long term, decreased PON1 enzyme activity can result in the development and progression of atherosclerosis and CAD [29, 30] due to increased reactive oxygen species and enhanced LDL oxidation [5, 31]. The role of PON1 in inhibiting of LDL oxidation is very important, and PON1 reduces oxLDL formation by 42–65% [32].

The rs854560 polymorphism has been analysed in populations of different ethnic origins. Our results are consistent with those previously reported. However, ethnic heterogeneity and different inclusion/exclusion criteria have led to inconsistent results and conclusions between studies. The T allele carrier state and TT homozygosity have previously been associated with CAD only in the Turkish population [20, 33]. In most other studies, including those examining North Indian [17], Brazilian Caucasian [34], and other Turkish populations [35, 36], the AA genotype was identified as a CAD risk factor. Furthermore, the results of other studies suggest a protective role of the T allele [18, 19] or a complete lack of association between the rs854560 polymorphism and CAD [11, 21, 37]. Inconsistencies between studies may be caused by different inclusion criteria of patients (e.g., stenosis ≥ 70% as a main criterion for inclusion [37]) or controls (e.g., the presence of hypertension, diabetes mellitus, and familial history of CAD in control subjects [18]).

Here, we also analysed the potential effect of the rs854560 polymorphism on the risk of CAD taking into account traditional risk factors. Our results indicate the presence of a strong synergistic effect between the TT genotype and cigarette smoking. Cigarette smoke contains a number of oxidizing compounds and is an important source of free radicals [38] which contribute to both the development of atherosclerosis and an increase in the incidence of cardiovascular events among smokers [39]. However, it must be noted that a limitation of the present study is the relatively small sample size and no reported dose of cigarettes. The results presented here should be confirmed in a larger group of patients, as well as in additional ethnic groups, and should include an additional information about nicotine exposure dose. Daily smoking dose may not only impact on cardiovascular disease but may also be related to the addictive behaviour [40]. It is possible that paraoxonase locus may be a determinant of smoking habit through metabolism of xenobiotics present in smoke [41].

Cigarette smoking is a potent inhibitor of PON1 enzymatic activity, making the effectiveness of PON1 in smokers particularly important [42, 43]. It was shown that cigarette smoke extract decreases PON1 activity by modifying the free thiol groups of the enzyme. It can be explained by the effects of several of the hundreds of chemical components of tobacco smoke including various reactive aldehydes as well as aromatic hydrocarbons [43]. Moreover, it is important that smoking may increase neutrophil myeloperoxidase activity [44], which has been shown to interact with HDL and to modulate paraoxonase 1 function [45]. The number of neutrophils was found to be higher in current or ex-smokers than in nonsmokers [46]. James et al. [47] found that the concentration and activity of PON1 in plasma were significantly lower in current smokers than in never smokers. In specific groups of US veterans [48], patients diagnosed with CAD [47] and inhabitants of the Spanish province of Gerona [42], a similar downward trend was observed. Furthermore, Mouhamed et al. [49] showed that, in smokers, the TT and AA genotypes presented the lowest and highest levels of PON1 activity, respectively. However, we did not observe differences in genotype distribution and allele frequencies between smokers and nonsmokers. These results are not consistent with previously described [49]. As in the case of association studies, these differences may also be due to differences in demographics, ethnic origin, matching criteria, and coexistence of other addictions. It is also possible that the observed differences result from chronic exposure to pollutants in Silesian Voivodeship, an industrial area where the study groups of the present work were recruited.

## 5. Conclusions

In conclusion, our results suggest that the rs854560 TT genotype may be a potent risk factor for CAD in the Polish population. Furthermore, TT homozygotes are particularly susceptible to the effects of smoking addiction, which decreases the activity of this protective and antiatherogenic enzyme. Functional studies support the idea that the TT genotype promotes the development of atherosclerosis and influences the manifestation of CAD, despite the conflicting results of case-control studies.

## Figures and Tables

**Table 1:** Clinical and biochemical characteristics of coronary artery disease patient (CAD) and blood donor (BD) groups.

Characteristics	CAD *n* = 248	BD *n* = 246	OR^∗^ (95% CI)	*p*
Age (years), mean ± SD	44.58 ± 5.98	43.58 ± 6.32	—	NS
Male gender, *n* (%)	173 (69.76)	175 (71.14)	0.94 (0.64–1.38)	NS
TC (mmol/L), mean ± SD	5.80 ± 1.35	5.09 ± 1.21	—	<10^−6^
HDL (mmol/L), mean ± SD	1.12 ± 0.37	1.45 ± 0.56	—	<10^−6^
LDL (mmol/L), mean ± SD	3.88 ± 1.18	2.99 ± 1.22	—	<10^−6^
TG (mmol/L), mean ± SD	1.86 ± 0.97	1.37 ± 0.72	—	<10^−6^
BMI ± SD	27.11 ± 4.24	26.15 ± 3.84	—	0.01
Familial history of CAD, *n* (%)	88 (50.57)	—	2.54 (2.25–2.86)^∗∗^	<10^−8^
Diabetes mellitus, *n* (%)	22 (8.98)	—	2.09 (1.90–2.29)^∗∗^	<10^−8^
Hypertension, *n* (%)	138 (57.26)	—	3.24 (2.77–3.78)^∗∗^	<10^−8^
Smoking, *n* (%)	146 (68.54)	67 (31.46)	3.82 (2.62–5.58)	<10^−7^

NS: not statistically significant; OR: odds ratio; SD: standard deviation; TC: total cholesterol; HDL: high-density lipoprotein; LDL: low-density lipoprotein; TG: triglycerides; BMI: body mass index. ^∗^Univariate analysis. ^∗∗^Risk ratio values (95% CI), univariate analysis.

**Table 2:** *PON1* rs854560 polymorphism genotype and allele frequencies in patient (CAD) and blood donor (BD) groups.

Genotype/allele	CAD *n* = 232 (%)	BD *n* = 221 (%)	Model of inheritance	OR (95% CI)	*p*
AA	96 (41.38)	94 (42.53)	Dominant versus AT + TT	0.95 (0.66–1.38)	NS
AT	103 (44.40)	109 (49.32)	Additive versus AA	0.93 (0.63–1.37)	NS
TT	33 (14.22)	18 (8.14)	Additive versus AA	1.80 (0.95–3.41)	NS
			versus AA + AT	1.87 (1.02–3.43)	0.04
AA + AT	199 (85.78)	203 (91.86)	Recessive versus TT	0.53 (0.29–0.98)	0.04
A	295 (63.58)	297 (67.20)	—	0.85 (0.65–1.12)	NS
T	169 (36.42)	145 (32.80)	—	1.17 (0.89–1.54)	NS

NS: not statistically significant; OR: odds ratio.

**Table 3:** Synergistic effect between the *PON1* rs854560 TT genotype and cigarette smoking (4 × 2 table).

TT homozygosity	Cigarette smoking	CAD (*n* = 228)	BD (*n* = 220)	OR (95% CI)
1	1	22	1	39.77 (5.26–300.70)
1	0	11	17	1.17 0.52–2.62)
0	1	117	61	3.46 (2.29–5.25)
0	0	78	141	1

OR: odds ratio; CAD: coronary artery disease (patient group); BD: blood donor (control group).

**Table 4:** Synergy index (with 95% CI) for interactions between *PON1* rs854560 TT genotype and traditional risk factors for CAD.

Traditional risk factors	SIM (95% CI)	SI (95% CI)	RERI (95% CI)	AP (95% CI)
Male gender	0.56 (0.12–2.57)	0.30 (0.02–4.53)	−1.32 (−9.89–1.27)	−0.84 (−7.26–0.53)
Overweight/obesity	2.67 (0.75–9.53)	10.04 (0.06–1568.05)	2.08 (−0.34–6.45)	0.63 (−0.41–0.86)
TC/HDL 5 : 1	0.68 (0.19–2.46)	1.16 (0.35–3.89)	0.71 (−4.18–10.27)	0.12 (−1.74–0.53)
TC (mmol/L)	0.53 (0.15–1.87)	0.86 (0.25–2.98)	−0.47 (−5.39–4.54)	−0.11 (−2.28–0.42)
LDL (mmol/L)	0.34 (0.09–1.24)	0.74 (0.25–2.22)	−1.56 (−9.40–5.04)	−0.29 (−2.66–0.36)
TG (mmol/L)	0.50 (0.14–1.73)	0.74 (0.21–2.60)	−0.92 (−4.98–3.72)	−0.26 (−2.63–0.32)
HDL (mmol/L)	1.10 (0.28–4.36)	1.77 (0.41–7.75)	2.06 (−2.42–13.96)	0.36 (−1.49–0.60)

**Table 5:** *PON1* rs854560 polymorphism genotype and allele frequencies in smoker and nonsmoker groups.

Genotype/allele	Smokers *n* = 201 (%)	Nonsmokers *n* = 247 (%)	Model of inheritance	OR (95% CI)	*p*
AA	80 (39.80)	108 (43.72)	Dominant versus AT + TT	0.85 (0.58–1.24)	NS
AT	98 (48.76)	111 (44.94)	Additive versus AA	1.19 (0.80–1.77)	NS
TT	23 (11.44)	28 (11.34)	Additive versus AA	1.11 (0.59–2.07)	NS
			versus AA + AT	1.01 (0.56–1.82)	NS
AA + AT	178 (88.56)	219 (88.66)	Recessive versus TT	0.99 (0.55–1.78)	NS
A	258 (64.18)	327 (66.19)	—	0.92 (0.69–1.21)	NS
T	144 (35.82)	167 (33.81)	—	1.09 (0.83–1.44)	NS

NS: not statistically significant; OR: odds ratio.

## References

[1] Quillen E. E., Rainwater D. L., Dyer T. D. (2012). Novel associations of nonstructural loci with paraoxonase activity. *Journal of Lipids*.

[2] Watson A. D., Berliner J. A., Hama S. Y. (1995). Protective effect of high density lipoprotein associated paraoxonase. Inhibition of the biological activity of minimally oxidized low density lipoprotein. *The Journal of Clinical Investigation*.

[3] Mackness M., Boullier A., Hennuyer N. (2000). Paraoxonase activity is reduced by a pro-atherosclerotic diet in rabbits. *Biochemical and Biophysical Research Communications*.

[4] Shih D. M., Xia Y. R., Wang X. P. (2000). Combined serum paraoxonase knockout/apolipoprotein E knockout mice exhibit increased lipoprotein oxidation and atherosclerosis. *The Journal of Biological Chemistry*.

[5] Aviram M., Billecke S., Sorenson R. (1998). Paraoxonase active site required for protection against LDL oxidation involves its free sulphydryl group and is different from that required for its arylesterase/paraoxonase activities: selective active of human paraoxonase alloenzymes Q and R. *Arteriosclerosis, Thrombosis, and Vascular Biology*.

[6] Gugliucci A., Menini T. (2015). Paraoxonase 1 and HDL maturation. *Clinica Chimica Acta*.

[7] Gaidukov L., Viji R. I., Yacobson S., Rosenblat M., Aviram M., Tawfik D. S. (2010). ApoE induces serum paraoxonase PON1 activity and stability similar to ApoA-I. *Biochemistry*.

[8] Navab M., Hama S. Y., Anantharamaiah G. M. (2000). Normal high density lipoprotein inhibits three steps in the formation of mildly oxidized low density lipoprotein: steps 2 and 3. *Journal of Lipid Research*.

[9] Primo-Parmo S. L., Sorenson R. C., Teiber J., La Du B. N. (1996). The human serum paraoxonase/arylesterase gene (*PON1*) is one member of a multigene family. *Genomics*.

[10] Richter R. J., Jarvik G. P., Furlong C. E. (2010). Paraoxonase 1 status as a risk factor for disease or exposure. *Advances in Experimental Medicine and Biology*.

[11] Cascorbi I., Laule M., Mrozikiewicz P. M. (1999). Mutations in the human paraoxonase 1 gene: frequencies, allelic linkages, and association with coronary artery disease. *Pharmacogenetics*.

[12] Akhmedova S. N., Yakimovsky A. K., Schwartz E. I. (2001). Paraoxonase 1 Met–Leu 54 polymorphism is associated with Parkinson’s disease. *Journal of the Neurological Sciences*.

[13] Ezzaher A., Mouhamed D. H., Mechri A. (2012). Association between bipolar I disorder and the L55M and Q192R polymorphisms of the paraoxonase 1 (PON1) gene. *Journal of Affective Disorders*.

[14] Arpaci A., Görmüs U., Dalan B., Berkman S., Isbir T. (2009). Investigation of PON1 192 and PON1 55 polymorphisms in ovarian cancer patients in Turkish population. *In Vivo*.

[15] Hussein Y. M., Gharib A. F., Etewa R. L., ElSawy W. H. (2011). Association of L55M and Q192R polymorphisms in paraoxonase 1 (PON1) gene with breast cancer risk and their clinical significance. *Molecular and Cellular Biochemistry*.

[16] Antognelli C., Mearini L., Talesa V. N., Giannantoni A., Mearini E. (2005). Association of CYP17, GSTP1, and PON1 polymorphisms with the risk of prostate cancer. *Prostate*.

[17] Agrawal S., Tripathi G., Prajnya R. (2009). Paraoxonase 1 gene polymorphisms contribute to coronary artery disease risk among north Indians. *Indian Journal of Medical Sciences*.

[18] Oliveira S. A., Mansur A. P., Ribeiro C. C., Ramires A. F., Annichino-Bizzacchi J. M. (2004). PON1 M/L55 mutation protects high-risk patients against coronary artery disease. *International Journal of Cardiology*.

[19] Aydin M., Gokkusu C., Ozkok E. (2009). Association of genetic variants in methylenetetrahydrofolate reductase and paraoxonase-1 genes with homocysteine, folate and vitamin B12 in coronary artery disease. *Molecular and Cellular Biochemistry*.

[20] Kaman D., İlhan N., Metin K., Akbulut M., Üstündağ B. (2009). A preliminary study of human paraoxanase and PON 1 L/M 55–PON1 Q/R 192 polymorphisms in Turkish patients with coronary artery disease. *Cell Biochemistry and Function*.

[21] Arca M., Ombres D., Montali A. (2002). PON1 L55M polymorphism is not a predictor of coronary atherosclerosis either alone or in combination with Q192R polymorphism in an Italian population. *European Journal of Clinical Investigation*.

[22] Niemiec P., Zak I., Wita K. (2007). The 242T variant of the CYBA gene polymorphism increases the risk of coronary artery disease associated with cigarette smoking and hypercholesterolemia. *Coronary Artery Disease*.

[23] Friedewald W. T., Levy R. I., Fredrickson D. S. (1972). Estimation of the concentration of low-density lipoprotein cholesterol in plasma, without use of the preparative ultracentrifuge. *Clinical Chemistry*.

[24] Knol M. J., VanderWeele T. J., Groenwold R. H., Klungel O. H., Rovers M. M., Grobbee D. E. (2011). Estimating measures of interaction on an additive scale for preventive exposures. *European Journal of Epidemiology*.

[25] Knol M. J., VanderWeele T. J. (2012). Recommendations for presenting analyses of effect modification and interaction. *International Journal of Epidemiology*.

[26] Zou G. Y. (2008). On the estimation of additive interaction by use of the four-by-two table and beyond. *American Journal of Epidemiology*.

[27] Leviev I., Deakin S., James R. W. (2001). Decreased stability of the M54 isoform of paraoxonase as a contributory factor to variations in human serum paraoxonase concentrations. *Journal of Lipid Research*.

[28] Garin M. C., James R. W., Dussoix P. (1997). Paraoxonase polymorphism Met-Leu54 is associated with modified serum concentrations of the enzyme. A possible link between the paraoxonase gene and increased risk of cardiovascular disease in diabetes. *The Journal of Clinical Investigation*.

[29] Jarvik G. P., Rozek L. S., Brophy V. H. (2000). Paraoxonase (PON1) phenotype is a better predictor of vascular disease than is *PON1*_192_ or *PON1*_55_ genotype. *Arteriosclerosis, Thrombosis, and Vascular Biology*.

[30] Zhou C., Cao J., Shang L. (2013). Reduced paraoxonase 1 activity as a marker for severe coronary artery disease. *Disease Markers*.

[31] Aviram M., Hardak E., Vaya J. (2000). Human serum paraoxonases (PON1) Q and R selectively decrease lipid peroxides in human coronary and carotid atherosclerotic lesions: PON1 esterase and peroxidase-like activities. *Circulation*.

[32] Zielaskowska J., Olszewska-Słonina D. (2006). The polymorphism of paraoxonase and its effects in physiological and pathological processes. *Advances in Clinical and Experimental Medicine*.

[33] Taşkıran P., Çam S. F., Şekuri C. (2009). The relationship between paraoxonase gene Leu-Met (55) and Gln-Arg (192) polymorphisms and coronary artery disease. *Archives of the Turkish Society of Cardiology*.

[34] Rios D. L., D'Onofrio L. O., Cerqueira C. C. (2007). Paraoxonase 1 gene polymorphisms in angiographically assessed coronary artery disease: evidence for gender interaction among Brazilians. *Clinical Chemistry and Laboratory Medicine*.

[35] Özkök E., Aydın M., Babalık E., Özbek Z., İnce N., Kara İ. (2008). Combined impact of matrix metalloproteinase-3 and paraoxanase 1 55/192 gene variants on coronary artery disease in Turkish Patients. *Medical Science Monitor*.

[36] Kocakap D. B., Doğru M. T., Şimşek V. (2015). The association of paraoxonase 1 gene L55M polymorphism with the extent and severity of coronary artery disease in the Turkish population and its dependence on gender. *Anatolian Journal of Cardiology*.

[37] Ahmad I., Narang R., Venkatraman A., Das N. (2012). Two- and three-locus haplotypes of the paraoxonase (PON1) gene are associated with coronary artery disease in Asian Indians. *Gene*.

[38] Park E. M., Park Y. M., Gwak Y. S. (1998). Oxidative damage in tissues of rats exposed to cigarette smoke. *Free Radical Biology and Medicine*.

[39] Salonen J. T., Malin R., Tuomainen T. P., Nyyssonen K., Lakka T. A., Lehtimaki T. (1999). Polymorphism in high density lipoprotein paraoxonase gene and risk of acute myocardial infarction in men: prospective nested case-control study. *British Medical Journal*.

[40] Shiffman S., Paty J. (2006). Smoking patterns and dependence: contrasting chippers and heavy smokers. *Journal of Abnormal Psychology*.

[41] Milnerowicz H., Kowalska K., Socha E. (2015). Paraoxonase activity as a marker of exposure to xenobiotics in tobacco smoke. *International Journal of Toxicology*.

[42] Senti M., Tomás M., Anglada R. (2003). Interrelationship of smoking, paraoxonase activity, and leisure time physical activity: a population based study. *European Journal of Internal Medicine*.

[43] Nishio E., Watanabe Y. (1997). Cigarette smoke extract inhibits plasma paraoxonase activity by modification of the enzyme’s free thiols. *Biochemical and Biophysical Research Communications*.

[44] Loke W. M., Lam K. M., Chong W. L. (2012). Products of 5-lipoxygenase and myeloperoxidase activities are increased in young male cigarette smokers. *Free Radical Research*.

[45] Huang Y., Wu Z., Riwanto M. (2013). Myeloperoxidase, paraoxonase-1, and HDL form a functional ternary complex. *The Journal of Clinical Investigation*.

[46] Parry H., Cohen S., Schlarb J. E. (1997). Smoking, alcohol consumption, and leukocyte counts. *American Journal of Clinical Pathology*.

[47] James R. W., Leviev I., Righetti A. (2000). Smoking is associated with reduced serum paraoxonase activity and concentration in coronary artery disease patients. *Circulation*.

[48] Jarvik G. P., Trevanian N., McKinstry L. A. (2002). Vitamin C and E intake is associated with increased paraoxonase activity. *Arteriosclerosis, Thrombosis, and Vascular Biology*.

[49] Mouhamed D. H., Ezzaher A., Mechri A. (2012). Effect of cigarette smoking on paraoxonase 1 activity according to PON1 L55M and PON1 Q192 R gene polymorphisms. *Environmental Health and Preventive Medicine*.

